# Chromium (III) Complexes of 4,5-diazafluoren-9-one Ligand as Potential Anti-proliferative Agents: Synthesis, Characterization, DNA Binding, Molecular Docking and *In-vitro* Cytotoxicity Evaluation

**DOI:** 10.22037/ijpr.2021.114685.14996

**Published:** 2021

**Authors:** Omolbanin Shahraki, Habib Ghaznavi, Niloufar Akbarzadeh-T, Sheida Shahraki, Roghayeh Sheervalilou, Tahere Kondori

**Affiliations:** a *Cellular and Molecular Research Center, Resistant Tuberculosis Institute, Zahedan University of Medical Sciences, Zahedan, Iran. *; b *Pharmacology Research Center, Zahedan University of Medical Sciences, Zahedan, Iran. *; c *Department of Pharmacology, School of Medicine, Zahedan University of Medical Sciences, Zahedan, Iran. *; d *Department of Chemistry, University of Sistan and Baluchestan, Zahedan, Iran.*

**Keywords:** Circular dichroism, Cyclic voltammetry, 4, 5-Diazafluoren-9-one, Phen-dione, Viscosity measurement

## Abstract

Three novel Cr(III) complexes, [Cr(dafone)_2_(H_2_O)_2_](NO_3_)_3_ (**1**), [Cr(opd) (dafone)_2_](NO_3_)_3_ (**2**) and [Cr (phen-dione) (dafone) (H_2_O)_2_].(NO_3_)_3_ (**3**) were synthesized and characterized by different techniques. Fluorescence spectroscopy, gel electrophoresis, viscosity measurement, and circular dichroism (CD) were applied to explore the interaction of Cr complexes with FS-DNA. The binding constant (K_b_) was obtained from UV–Vis measurements. The obtained results exhibited the effective binding of target complexes to DNA double-strand. The fluorescence data appraised both binding and thermodynamic constants of complexes-DNA interactions. The measured thermodynamic factors (∆S˚, ∆H˚, ∆G˚) revealed that hydrogen bonding and van der Waals forces for DNA- Cr(III) complexes bear the most important roles. As well, the Stern–Volmer quenching constants (K_sv_) and the binding constants (K_b_) of synthesized compounds and DNA were calculated. The results of thermodynamic parameters showed that the binding of synthesized Cr(III) compounds to DNA was driven mainly through hydrogen bonds and van der Waals interactions. Viscosity measurement results showed that increasing the concentration of synthesized compounds, did not make any major changes in specific viscosity of FS-DNA. The data of viscosity and circular dichroism (CD) support the groove binding mode.

## Introduction

**Figure 1:**
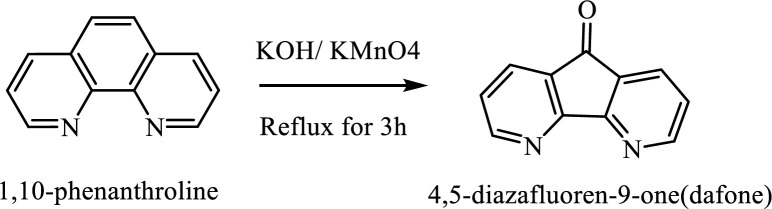
Synthesis of dafone

Bidentate heterocyclic nitrogen-containing bases, such as 1,10-phenanthroline (phen), and its analogs such as 1,10-Phenanthroline-5,6-dione and synthetic derivatives as 4,5-Diazafluoren-9-one, are important as chelating agents in coordination chemistry (1). One of them is dafone (4,5-Diazafluoren-9-one) (Figure 1) that has been reported in the literature (2, 3), and compared with phen and bipyridine (4). Dafone as one of 1, 10-phenanthroline derivatives, has been reported as a premier ligand with various coordination chemistry utilizations. Dafone, on the other hand, forms fewer transition metal complexes than 1, 10-phenanthroline. 

Dafone complexes of Ru (II) and Eu (III) have potential functions in the optical field and have been thoroughly investigated (5, 6). First-row transition metal compounds comprising poly pyridyl moiety, not only because of pharmacological effects but also due to various applications as a molecular scaffold in electrochemistry, as catalysts and also, as a constituent for metal dendrimer synthesis (7, 8). Bearing three single electrons, chromium (III) complexes are paramagnetic. This topic has been confirmed in all the single-core complexes (9, 10). Chromium (III) complexes are stable to oxidation in water and a lot of complexes of chromium (III) are octahedral in structure (11, 12) Several 1,10-phenanthroline derivatives like 4,5-diazafluoren-9-one (dafone) have gained consideration possibly as a result of their DNA intercalation properties (13). some attempts were carried out to obtain the chemical and physical characteristics of the π system by chemical alteration of phen ligand (14).

Based on the importance of these compounds and our attention to the chemistry of phenanthroline analogs, herein, we describe the synthesis of the three chromium (III) compounds with the formula [Cr(dafone)_2_(H_2_O)_2_](NO_3_)_3_** (1)**, [Cr(opd)(dafone)_2_](NO_3_)_3_ **(2)** and [Cr(phen-dione)(dafone)(H_2_O)_2_](NO_3_)_3 _**(3).** These compounds were characterized using FT-IR, UV-Vis spectroscopies, elemental analysis, and cyclic voltammetry method (CV). In this study, Cr(III) compounds include phen ligands, as new DNA binding agents have been introduced. Then, ﬂuorescence spectroscopy, viscosity measurement, and circular dichroism (CD) have been applied for investigating the interaction of synthesizSed complexes with DNA. Notably, we have investigated the FS-DNA cleaving capability of chromium (III) complexes using the gel electrophoresis method. The cytotoxicity evaluation of synthesized compounds proposed a high potency against cancerous A549 and KB cells in comparison to control following treatment with any of the complexes **1** and **2 **while compound **3** exhibited lower potency toward these cell lines. Molecular docking results confirmed efficient binding of the target compounds to DNA structure and confirmed groove mode of binding. 

## Experimental

*Materials and methods*

Chemicals and solvents were purchased from Merck company and used without any puriﬁcation. 4,5-Diazafluoren-9-one was prepared from 1-10 phenanthroline using a reported procedure (5). FS-DNA was purchased from Sigma Chemical Company. Shimadzu-470 spectrometer was employed to acquire Infrared spectra (400- 4000cm^-1^) as one percent dispersions in KBr pellets. ^1^H NMR spectra were achieved on a 300-MHz spectrometer using DMSO-d6 as solvent and tetramethylsilane as the internal standard. a Heraeus CHN–O Rapid analyzer was applied for Elemental analysis. The UV–Vis spectra were obtained on a Shimadzu 2100 spectrometer. Cyclic voltammograms were acquired by SAMA 500. The fluorescence data were evaluated by a PerkinElmer, LS-45.

RPMI 1640 and DMEM-F12 media, L-glutamine, penicillin, and streptomycin were procured from Biosera (Austria). Fetal bovine serum (FBS) was purchased from Gibco (USA). Cisplatin and 3-(4,5-dimethylthiazol-2-yl)-2,5-diphenyltetrazolium bromide (MTT) were obtained from EBEWE Pharma (Austria) and Sigma Aldrich (Germany) respectively. 

*Synthesis of dafone ligand (4,5-Diazafluoren-9-one)*

A mixture of 1-10 phenanthroline hydrate (2.0 g, 10 mmol) and KOH (1.0 g) was added to 100 mL H_2_O in a flask and heated until boiling. Then, 50 mL hot aqueous solution of potassium permanganate (5 g KMnO_4_) to this solution and refluxed at 100 °C for three hours. During the addition, the solution was stirred. Then the reaction content was filtered to eliminate the manganese dioxide precipitates. The orange deposit was extracted with chloroform and was allowed to slowly evaporate at room temperature. Yellow needle-like crystals were separated 3 days later. The synthesis procedure is illustrated in Figure 1 (15).

*Synthesis of complex [Cr(dafone)*_2_ *(H*_2_ *O)*_2_ *](NO*_3_ *)*_3_ *(1)*

**Scheme 1:**
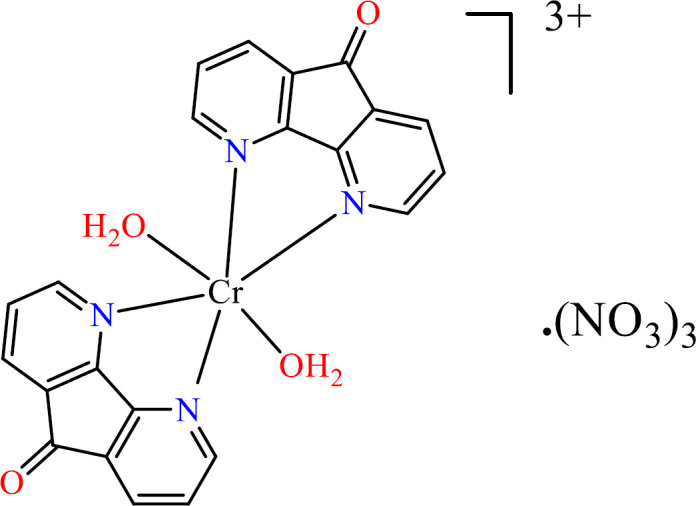
Structure of [Cr(dafone)_2_(H_2_O)_2_](NO_3_)_3_ **(1)**.

A solution of 4,5-Diazafluoren-9-one (dafone) (0.182 g, 1 mmol,) in 10 mL H_2_O was added to a 10 ml aqueous solution of Cr(NO_3_)_3 _·9H_2_O (0.200 g, 0.5 mmol). The resulting solution was stirred and refluxed at 100 °C for 24 h. The blueish product, [Cr (dafone)_2_(H_2_O)_2_](NO_3_)_3 _(Scheme 1) was ﬁltrated and air-dried. Anal. Calc: C, 44.45; H, 2.84, N, 16.59; Found: C, 44.38; H, 2.53; N, 16.13.

*Synthesis of complex [Cr(opd)(dafone)(H*_2_ *O)*_2_ *](NO*_3_ *)*_ 3_ *(2)*

**Scheme 2:**
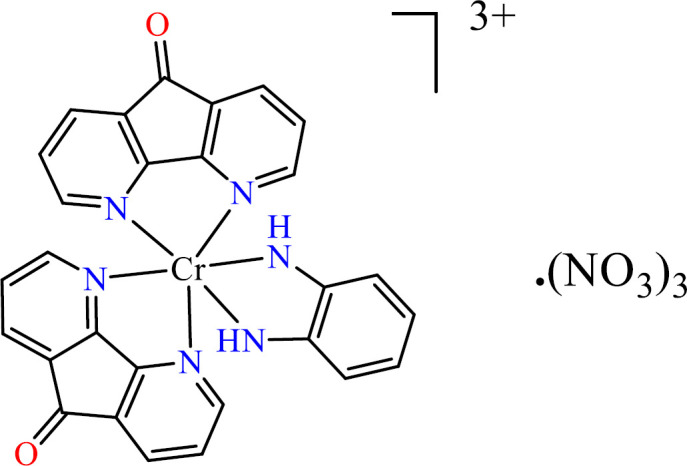
Structure of [Cr(opd)(dafone)_2_](NO_3_)_3_ **(2)**.

To an ethanolic solution (10 mL) of 1, 2-Phenylenediamine (opd), (0.054 g, 0.5 mmol) Cr (NO_3_)_3 _·9H_2_O (0.200 g, 0.5 mmol) in10 ml H_2_O was added. Then 10 mL aqueous solution of dafone (0.5 mmol, 91 mg) was added. The obtained solution was stirred and refluxed at 100 °C for 24 h. Then, the solution was left to evaporate slowly at room temperature to give a bluish-purple powder (scheme 2). Anal. Calc: C, 47.26; H, 3.11, N, 17.69; Found: C, 47.25; H, 3.21; N, 17.37.

*Synthesis of complex [Cr(phen-dione)(dafone)(H*_2_ *O)*_2_ *](NO*_3_ *)*_3_ *(3)*

**Scheme 3:**
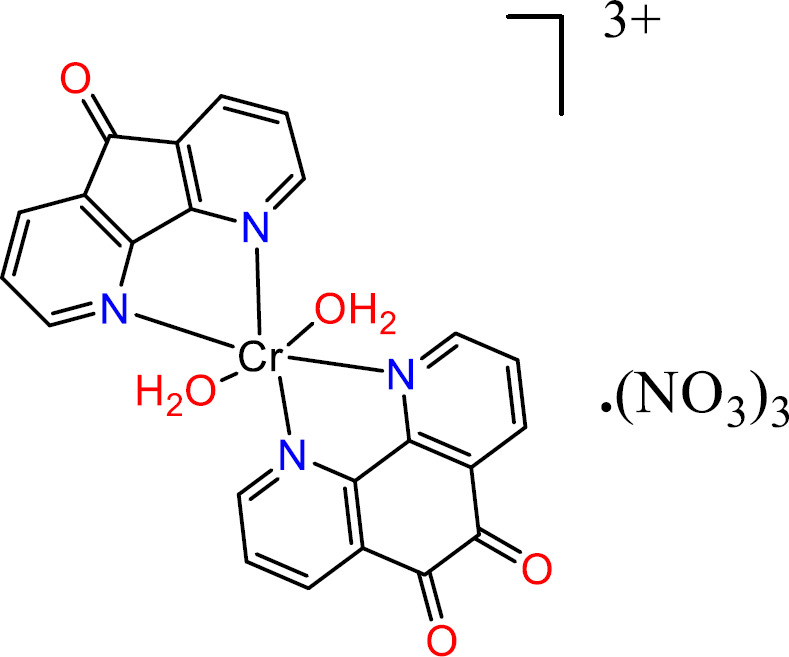
Structure of [Cr(phen-dione)(dafone)(H_2_O)_2_](NO_3_)_3_**(3)**.

To a warm ethanolic solution (10 mL) of 1, 10-Phenanthroline-5,6-dione (phen-dione), (0.105 g 0.5 mmol) of Cr(NO_3_)_3_·9 H_2_O (0.200 g, 0.5 mmol) in 10 ml H_2_O was added. After stirring for 3h at room temperature, (0.5 mmol, 91 mg) of dafone dissolved in 10 mL of warm ethanol was added dropwise. The obtained solution was mixed and refluxed at 100 °C overnight and air-dried at room temperature to give green-black powder (scheme 3). Anal. Calc: C, 41.45; H, 2.42, N, 14.71; Found: C, 41.25; H, 2.53; N, 15.1

*DNA binding experiments*

The DNA stock solution (2 mg/mL) was freshly made in distilled water. The value of pH was set at 7.2. H_2_O was used as a solvent for the preparation of Cr(III) complexes Stock solutions (1 × 10^–3^ M). To investigate the interaction of the synthesized Cr(III) complexes with FS-DNA, the electronic absorption titration test was carried out. In these tests, constant concentrations of the synthesized complexes **1**, **2,** and **3** (3.7 × 10^-5^, 6.8 × 10^-5^, 9.8 × 10^-5^ M) were titrated with different concentrations. 

Fluorescence spectra were obtained in different concentrations of FS-DNA. In this study, the concentration of the Cr(III) complexes was remained constant while changing the DNA concentration. In gel electrophoresis experiments the samples were blended with loading buffer, DNA, and methylene blue and were mixed. An agarose gel was used to load the solution. Finally, the Peaks were photographed after being irradiated with UV light.

*Cell culture*

In this study, two cancerous cell lines: human lung adenocarcinoma cell line (A549), oral squamous carcinoma cell line C152 (KB) and normal HUVEC line (human umbilical vein endothelial cells) were used. The target cell lines were purchased from the National Cell Bank of Iran (Pasteur Institute). Dulbecco’s modified Eagle’s medium (DMEM) supplemented with 15% fetal bovine serum was used as culture media for A549 and HUVEC cells. KB cells were cultures in RPMI-1640 containing 10% fetal bovine serum. The culture media were treated with penicillin (100 U/mL), and streptomycin (100 μg/mL) in humidified conditions with 5% CO_2_ at 37 °C. 

*Cytotoxicity evaluation (MTT) assay*

The anti-proliferative potential of synthesized compounds was evaluated by conversion of MTT (3-(4, 5-dimethyl thiazol-2yl)-2, 5-diphenyl tetrazolium bromide) to insoluble formazan. Based on previously reported protocols (16), the cells were seeded (as triplicates) in 96-well cell culture plates (5 × 10^3 ^cells/well) and cultivated overnight in the culture medium under standard conditions (95% humidified air, 37 °C, 5% carbon dioxide). After incubation, diverse concentrations of compounds were added and the cells were further incubated for 72 h. Then, 20 μL of MTT solution (5 mg/mL) in phosphate-buffered saline (PBS) was added to each well. After incubating for 3 h at 37 °C, the supernatant was removed and formazan crystals were dissolved by adding 200 μL dimethyl sulfoxide (DMSO). The absorbance was measured using a Stat Fax 2100 microplate reader at 570 nm. The cytotoxicity results were calculated as IC_50_ values using Curve Expert (version 1.4) software. The data are the mean value of three replications in triplicate. 

*Molecular docking simulations*

FFT-based (fast Fourier transform) HEX 8.0.0 software was applied for molecular docking studies. The DNA structures (PDB codes: 1BNA and 1DNE) in PDB format were retrieved from the RCSB protein data bank (http://www.rcsb.org/). All heteroatoms such as water molecules and cognate ligands were eliminated (17, 18). The synthesized compounds were energetically minimized using Hyperchem 7.5 program. The different parameters for docking have remained as default (correlation type: shape only, FFT mode: 3D, grid dimension: 0.6, receptor range: 180, ligand range: 180, twist range: 360, and distance range: 40). The PyMOL Molecular Graphics System, Version 1.2r3pre, Schrödinger, LLC software was used for visualization of the results. 

## Result and Discussion

The reaction of [Cr(NO_3_)_3_·9H_2_O)] with dafone, opd and phen-dione led to the formation of target complexes. At room temperature, these compounds are air-stable. The results of the elemental analysis were compatible with their formula. The coordination method of the ligands in the complexes was investigated using spectroscopic methods.

*FT-IR spectra of complexes (* ***1*** *), (* ***2*** *), and (* ***3*** *)*

**Figure 2:**
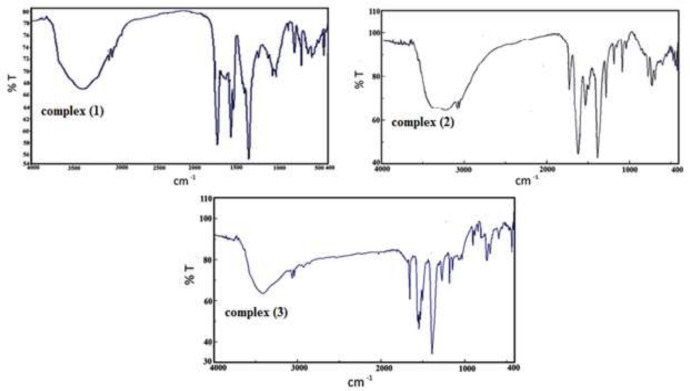
FT-IR spectra of complexes (**1**), (**2**) and (**3**).

IR spectroscopy is a useful strategy for the characterization of transition metal complexes (Figure 2). The IR spectrum of 4,5-Diazafluoren-9-one consists of broadband at 2900-3100 cm^-1 ^associated with ν(C-H) stretching frequency. The detected band at 1719 cm^-1 ^is assigned to ν (C=O) vibration (19). The band at 1380 cm^-1 ^is associated with NO_3_^-^ ion (13, 20, 21). The observed bands at 1571 cm^-1^, 1596 cm^-1^ and 1565 cm^-1 ^are assigned to ν(C = N) and ν(C = C) vibrations in Pyridine rings (22). The bands in the range 750-1000 cm^-1 ^are attributed to the bending vibration of ν(=C-H) (23, 24). The vibrational bands at 3088 cm^-1^ in complexes are assigned to ν(C–H). The band at 420 cm ^−1^ is assigned to ν(Cr–N). The broadband around 3500 cm^-1 ^is associated with stretching band ν(O-H) in the H_2_O ligand. 

In complex (**3**) the stretching band at 1000-1350 cm^-1 ^at the complex is assigned to ν(C-N) and the bands observed between 1480-1560 cm^-1 ^are assigned to ν(C = N) and ν(C = C) vibrations in Pyridine rings (25) The vibrational bands between 2900-3100 cm^-1^ in the complex are assigned to v(C–H). The band observed at 1632 cm^-1 ^is assigned to v(C = O) and phen-dion ligand (19). Complex **(3) **displays a band at 430cm^−1^. The broadband at 3500 cm^-1^ is related to stretching ν (O-H) in the H_2_O ligand (19).

*UV-Vis spectra of complexes* *(****1****), (****2****) and (****3****)*

**Figure 3:**
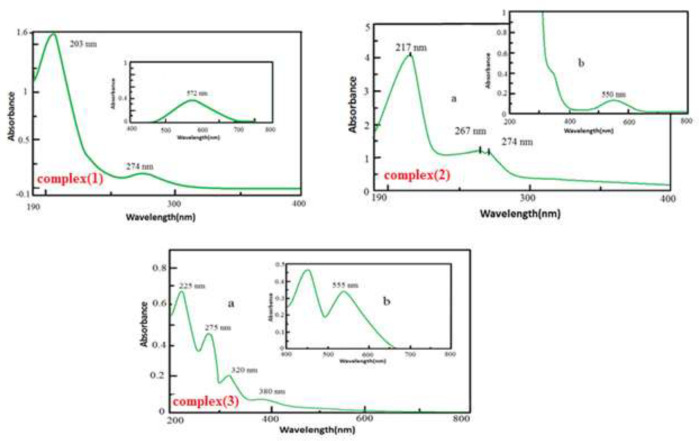
UV–Visible spectra of complexes (**1**), (**2**), and (**3**) in water (1 × 10^-3^ M in Vis (b) and 1 × 10^-5^M in UV (a) region).

The electronic spectrum of [Cr(dafone)_2_(H_2_O)_2_](NO_3_)_3_ in an aqueous solution bears some absorption bands in the UV and visible region (Figure 3). The peak at 572 nm, in the visible region, is attributed to ^4^A_2g_→^4^T_2g_ as d–d transition. Strong UV bands at about 203 nm and 274 nm were assigned to (π→π*, n→π*) dafone intra-ligand transitions. According to the literature transition in 274 nm is related to ^4^A_2_g → ^4^T_1_g _(F)_ (26, 27).

In complex (**2**), the band at 550 nm in the visible region is attributed to the ^4^A_2g_→^4^T_2g_ transition that is assigned to the d–d transmission electron. Strong UV bands at approximately 217 nm and 267 nm were referred to dafone intra-ligand (π→π*, n→π*) transitions. According to the literature ^4^A_2_g → ^4^T_1_g transmission (F) is placed in the area 274 nm (26, 27).

In complex (**3**), the band at 555 nm in the visible region is attributed to ^4^A_2g_→^4^T_2g_ transition that shows d–d transmission electron. Strong UV peaks at about 200-300 nm were allocated to dafone intra-ligand (π→π*, n→π*) transitions and phen-dione. Absorption bands at 225, 275, 320, and 380 nm are related to intra-ligand (π→π*, n→π*) transitions in dafone and phen-dione(5, 27). Transmission of chromium (III) is not divisible because of overlap with the intra-ligand transmissions and LMCT transmissions.

^1^ *H-NMR study*

**Figure 4:**
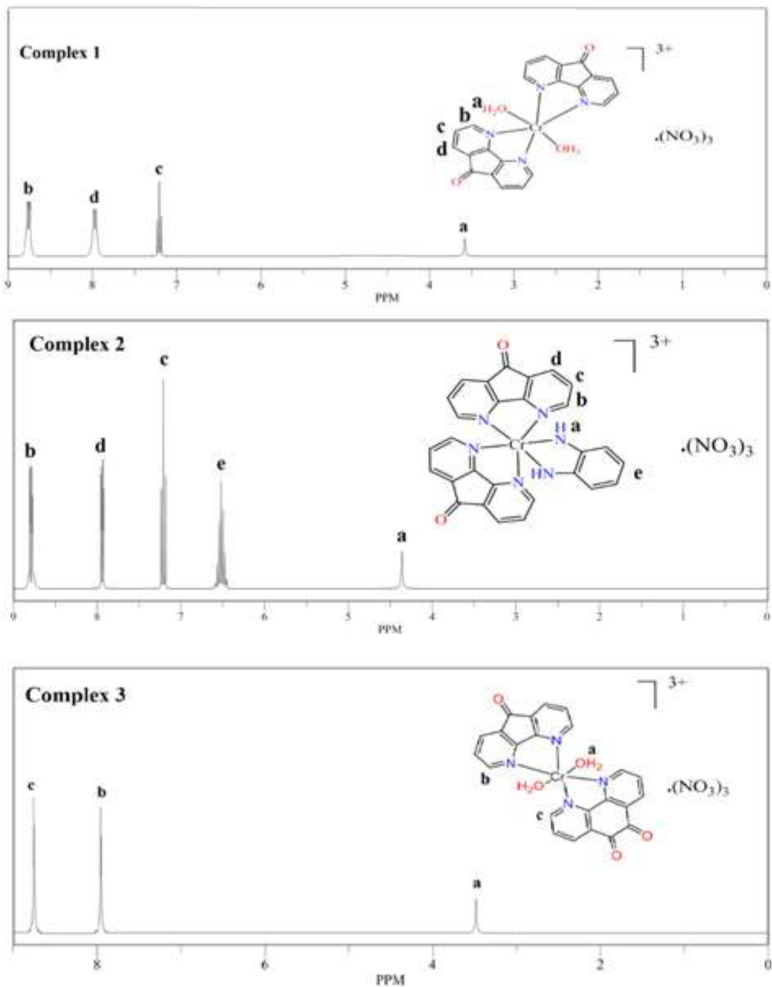
^1^HNMR spectra of complexes **(1),** **(2)** and **(3)** in DMSO-d_6_ at 25 °C

^1^H NMR spectra of complexes **(1),** **(2)** and **(3)** in DMSO-d_6_ at 25 °C are presented in Figure 4. The ^1^H-NMR spectrums of the complexes were analyzed by comparing of their ligands. The signals in the free ligands appear in 6.6-8.5 ppm. 

It was also the signals of protons near to paramagnetic center (Cr^III^) in complexes **(1),** **(2),** and **(3)** that have been less broad and appear in the low field. The signal at 3.6 ppm and 3.4 ppm attributed to H_2_O molecules in the complex **(1) **and** (3) **respectively (30). Amine hydrogen atoms of the opd ring in complexes **(2)** were observed at 4.7 ppm.

*Cyclic voltammetry of complexes (1), (2) and (3)*

The cyclic voltammogram for complex (**1**) was achieved at 25 °C in DMF solvent containing 0.1M TBAH as secondary electrolyte with a scan rate of 500 mvs^-1^. Complex (**1**) shows quasi reversible reduction waves at -1.2 V and -0.85V versus Fc/ Fc+ couple attributed to reduction of Cr^+3^/Cr^+2^. Waves at negative potential -1.58 V are assigned to the reduction of ligand (8, 26, 27).

Comparing voltammogram of free ligand and complex [Cr(opd)(dafone)(H_2_O)_2_](NO_3_)_3_, can be attribute to oxidation and reduction of 1,2-Phenylenediamine (0.72 v, 0.55 v) (28), complex (**2**) shows quasi reversible reduction waves between (-1.25 v, -1.32 v) against the Fc/ Fc^+^ pair assigned to reduction of Cr^+3^/ Cr^+2^ and waves at negative potential (-0.25v, -0.45v) assigned to reduction of dofane (27).

**Figure 5:**
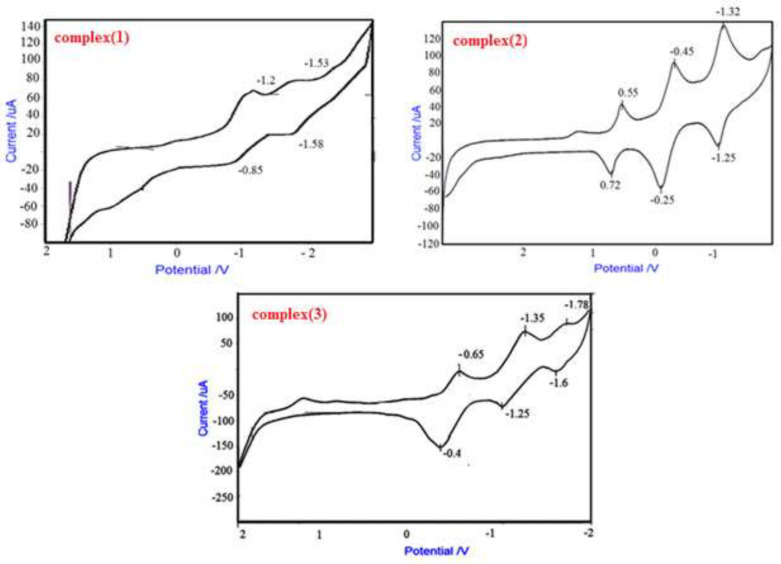
Cyclic voltammetry of complexes (**1**), (**2**) and (**3**), in DMF solvent, 0.1 M TBAH as a supportive electrolyte

In this complex three oxidation-reduction peaks can be seen. Comparing voltammogram of free ligand and complex that were acquired at 25 °C in H_2_O solution comprising 0.1M TBAH as secondary electrolyte with a scan rate of 500 mvs^-1^, peaks at -1.25v and -1.35v can be attributed to oxidation and reduction of free phen-dione, and -0.4 v and -0.65 v to dafone (24). Complex (3) displays quasi reversible reduction waves at -1.78 v and -1.6 v against Fc/ Fc^+^ couple assigned to reduction of Cr^+3^/ Cr^+2^ (Figure 5) (29).

*DNA Binding Investigations*

*DNA binding experiments*

The DNA binding study was carried out using a freshly prepared DNA solution in double-distilled water. The pH value was set at 7.2. Stock solution of Cr(III) complex (1 × 10^–3^ M) was prepared in H_2_O as the solvent. The interactions of the target complexes Cr(III) with DNA, the electronic absorption titration test was carried out. For this purpose, constant concentrations of the synthesized metal complexes (3.7 × 10^-5^, 6.8 × 10^-5^, 9.8 × 10^-6^ M for complexes **1**, **2,** and **3**, respectively) were titrated with adding the amount of DNA solution ([DNA] = (7-42), (5-50) and (10-40) µM for complexes **1**,**2** and **3**, respectively). The stability of complexes in an aqueous medium was examined comparing their UV–Vis spectra at 12 h intervals and the complexes exhibited high levels of stability. 

To achieve equilibrium, DNA-Cr(III) complex solutions were prepared 10 minutes earlier than recording the UV–Vis spectra. Then, the FS-DNA solution was added and compared to the blank DNA solution. Fluorescence spectra were obtained for different concentrations of FS-DNA solution. This study,

Was performed at a constant concentration of synthesized compounds (1.7 × 10^-6^, 2.1 × 10^-5^, 1.0 × 10^-7^ M for complexes **1**,**2** and **3**, respectively) and various concentrations of FS-DNA (0-24), (0-70) and (0-32) mΜ. The excitation wavelength for complexes **1**, **2,** and **3** was recorded at 310, 351, and 378 nm at different temperatures levels (303, 298, and 293 K). also, gel electrophoresis tests were carried out. Thus, some samples with a constant concentration of DNA and several concentrations of complexes were remained at about 1-hour in a solution of Tris–buffer. 

The target samples were mixed with 4 μL of a loading buffer, 5 μL of the DNA solution, and methylene blue. After shaking, the mixture was loaded onto an agarose gel, after running the electrophoresis at 100 V for 30 min and photographed after exposure to UV irradiation.

*Viscosity titration measurements*

Viscosity tests were performed by an Anubbelohde viscometer immersed in a stated water bath at a constant temperature (27.0 ± 0.1 °C) for three synthesized complexes. The measurement was repeated about three times, so the mean values were reported.

*CD spectral measurements*

The corresponding circular dichroism spectra of FS-DNA in the absence and presence of chromium (III) compounds were obtained at 25 °C at 190 -330 nm. The circular dichroism spectra were recorded at a scan rate of 200 nm.min^-1^ and a 1s response period, the results were obtained each 0.2 nm from 190 to 330 nm after 3 repletion.

*Electronic absorption spectra*

**Figure 6:**
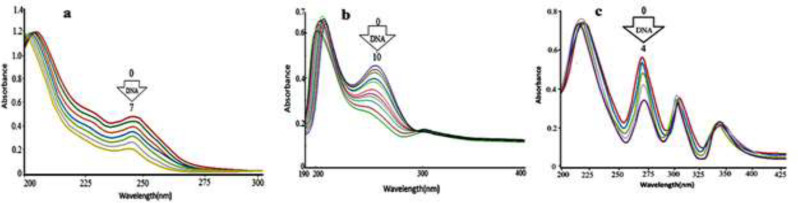
UV-Vis spectra of a) complex **1** whitout (line 0) and with different [DNA] (line 1-7) [Complex] = 3.7 × 10^-5^ M, [DNA] = (7-42 μM). b) Complex **2** whitout (line 0) and with different [DNA] (line 1-10 [Complex] = 6.8 × 10^-5^ M, [DNA] = (5-50 μM). c) Complex **3** whitout (line 0) and with different [DNA] (line 1-4 [Complex] = 9.8 × 10^-5^ M, [DNA] = (10-40 μM).

UV-Vis spectral data were applied to define the strength and modes of small molecule binding to DNA. The complex **1 **related spectra in the constant concentration of 3.7 × 10^-5^M with different DNA concentrations (1.4 × 10^-4^ M, 0-42 μL) are shown in Figure 5 indicating that increasing in FS-DNA concentrations diminish the absorption intensity. The UV-Vis absorptions were recorded for synthesized complexes in interaction with FS-DNA (Figure 6). The results approve the interaction of complex (**1**) and DNA.

**Figure 7:**
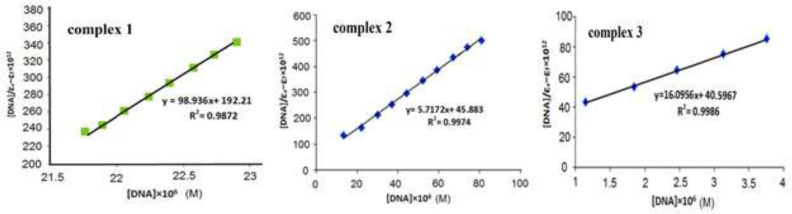
The curve of [DNA]/(εa-ε_f_) × 10^12^ versus [DNA] × 10^6^

The binding constant (K_b_) was calculated by means of [DNA]/(ε_a_-ε_f_)*10^12^ versus [DNA]*10^6^ plot that is derived from the slope-to-intercept ratio (Figure 7). The corresponding K_b _value from UV-Vis (5.1 × 10^5^) is less than the K_b _value associated with the traditional intercalator Ethidium bromide (1.4 × 10^6^) (30-32). 

Ethidium bromide has a planar system that can easily bind to DNA and simulate the change in the DNA conformation. Lastly, it is predictable that DNA-compound **1** binding mode was not intercalation. Also, the hyperchromic shift specifies the absence of intercalation mode (32, 33). Finally, groove binding is the interaction mode of DNA- complex. Consequently, the interaction of DNA with complex **1** is aided by hydrogen bonding. The same procedure was conducted in the case of complexes **2** and **3** and the K_b _values are presented in Table 1.

*Fluorescence investigations*

The fluorescence technique was used to investigate the interactions of DNA macromolecules with synthesized Cr(III) complexes. Electronic absorption analyses for complexes **1**, **2** and **3** show that the interactions are non-intercalative. Intercalation is also undesirable as a result of ligand steric constrain surrounding the Cr(III) ion. As a result, the most likely mode of interaction between complexes 1, 2, and 3 and FS-DNA is groove binding.

**Figure 8:**
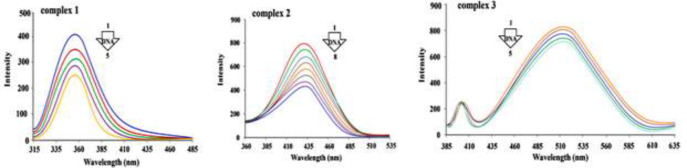
Complexes **1, 2 **and** 3 **(1.7 × 10^-6^, 2.1 × 10^-5^, 1.0 × 10^-7^ M) emission spectra with adding amount of DNA, at 298 K. complex **1**) the concentration of FS-DNA increases from zero (line 1) to 24 µM (line 5); complex **2**) DNA concentration increases from zero (line 1) to 70 µM (line 8); complex **3**) concentration of DNA increases from zero (line 1) to 32 µM (line 5).

Figure 8 illustrates the emission spectra of the synthesized compounds in interaction with varying concentrations of DNA. The excitation wavelength for the target complexes 1, 2 and 3 were at 310, 351 and 378 nm, respectively.

Figure 7 depicts that emission spectra intensity decreases with the increase in DNA concentration. data indicate that the fluorescence of the complexes is quenched by DNA solution.

The acquired fluorescence data were used for exploring the interactions. The quenching mechanism can be either static or dynamic. The Stern-Volmer equation (F_0_/F = 1 + *K*_sv_ [Q] = 1+ *k*_q_ τ_0 _[Q]) was applied for the analysis of fluorescence quenching data.

**Figure 9:**
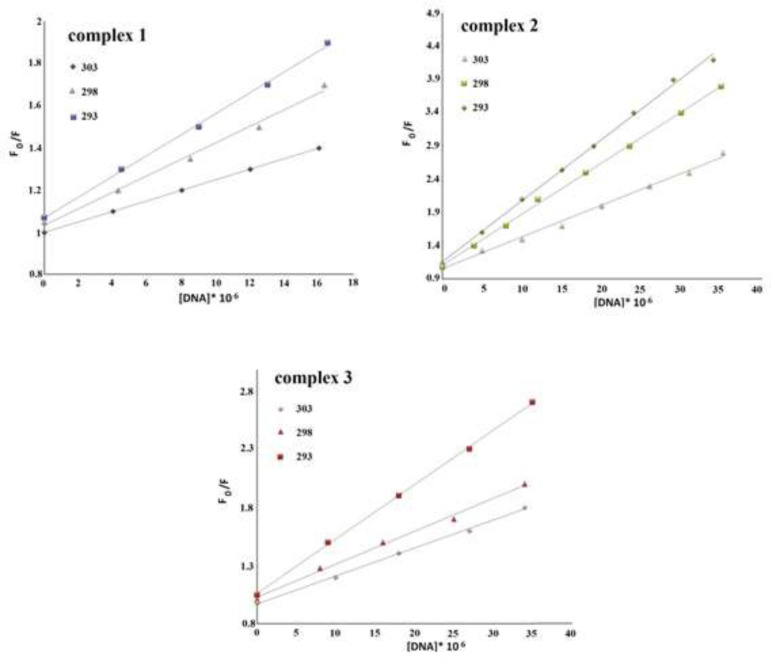
The Stern-Volmer graphs of Cr(III) complexes of **1**, **2** and **3** at 303, 298 and 293 K

In the above-mentioned equation, F_0 _stands for fluorescence intensities of the synthesized compound when there are not any quencher species (DNA),

Compound fluorescence intensities exposed to various quencher concentrations are shown by F,

Stern–Volmer quenching coefficient is shown by *K*_sv_*,* [Q] shows the quencher concentration, *k*_q_ stands for to the bimolecular quenching level constant and lastly, τ_0_ refers to the fluorophore lifetime (τ_0_ = 10^−8^ s) (34). The Stern–Volmer quenching constant (*K*_sv_) was calculated based on the slope of F_0_/F vs [DNA] plot (Figure 9) and *k*_q_ from *K*_sv_ / τ_0_ at different temperatures of 303, 298 and 293 K. The data are summarized in Table 1. 

The results in Table 1 indicate that the decrease in *k*_q_ and *K*_sv_ with growing temperature and *k*_q_ value is more than 2.0 × 10^10^ M^-1^s^-1^, showing that the quenching mechanism is static (35, 36). 

The equation of {log ((F_0_ – F)/F) = log *K*_b_+*n*log[Q]} (37) is used to calculate the binding constant (*K*_b_) and binding sites (n) values from the obtained data from fluorescence measurements.

**Figure 10:**
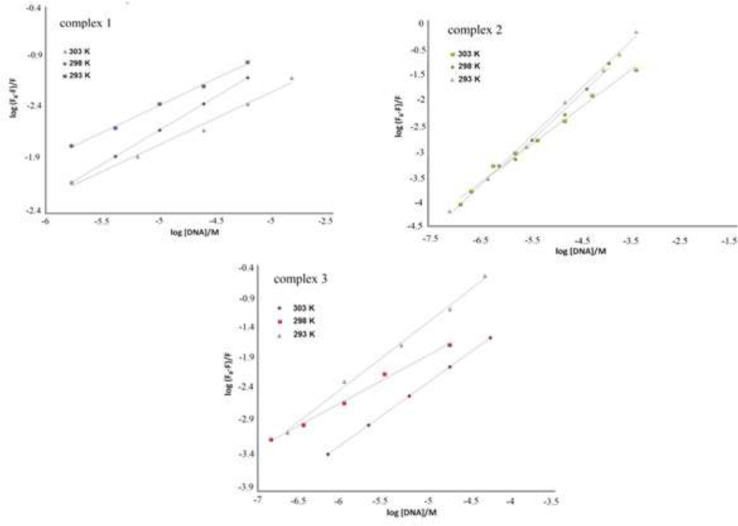
log ((F_0_ – F)/F) *vs*. log ([DNA]/μM) for the fluorescence titration of Cr(III) complexes **1**, **2** and **3** with DNA at different temperatures (303, 298 and 293 K).

Based on this equation, n and K_b_ were calculated from the intercept and slope of log ((F_0_ – F)/F)) *vs*. log ([DNA]/μM) graph (Figure 10), respectively. Table 1 shows that as the temperature rises, the amount of K_b_ decreases, suggesting that the interaction between DNA and Cr(III) compounds may occur through an exothermic mechanism (38). The binding sites number is approximately one that means Cr(III) complexes have one independent active site on the DNA double-strand (39, 40).

*Fluorescence data and thermodynamic factors*

**Figure 11:**
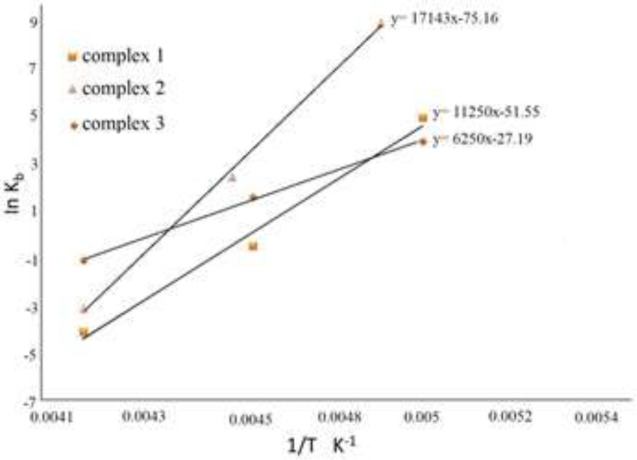
The van’t Hoff curve in the interactions of complexes **1**, **2** and **3**; with DNA at various temperatures (303, 298 and 293 K).

It is important to make a distinction between standard entropy (*ΔSº*), enthalpy (*ΔHº*) and Gibbs free energy (*ΔGº*) changes to comprehend the thermodynamic reaction between the specified molecules and FS-DNA. The thermodynamic parameters are applied for determining the nature of interactions. *∆Sº* and *∆Hº* were acquired utilizing *K*_b _values at various temperatures and the van’t Hoff equation (ln* K*_b_ = -*ΔHº*/R*T* + *ΔSº*/R) (32, 41). The titled parameters were determined using ln* K*_b_ vs 1/T curve (Figure 11) that slope (–*ΔH*º/R), and the intercept (*ΔSº*/R), are related to *∆Hº* and *∆Sº *respectively. The *ΔG*º factor can be obtained from the equation of *ΔGº* = *ΔHº* – *TΔSº* = –R*T*ln*K*_b_. The data are provided in Table 1.

A minimal or zero value of ∆Hº and positive value of *∆Sº* represent electrostatic interactions, while negative *∆Sº* and ∆Hº values indicate hydrogen bonding and van der Waals interactions, and positive *∆Sº* and ∆Hº represent hydrophobic interactions. As a result, the negative *ΔH* º and *ΔSº* values (shown in Table 1) prove that Cr(III) complexes in DNA grooves are stabilized by hydrogen bonding and van der Waals interactions. The negative numbers of *ΔGº*, on the other hand, indicate spontaneous phenomena (41). 

Lastly, the exothermic interaction of Cr (III) complexes and DNA is shown by the negative quantities of *ΔHº*. As the temperature rises, the value of *K*_b_ decreases (Table 1).

*Viscosity measurement*

**Figure 12:**
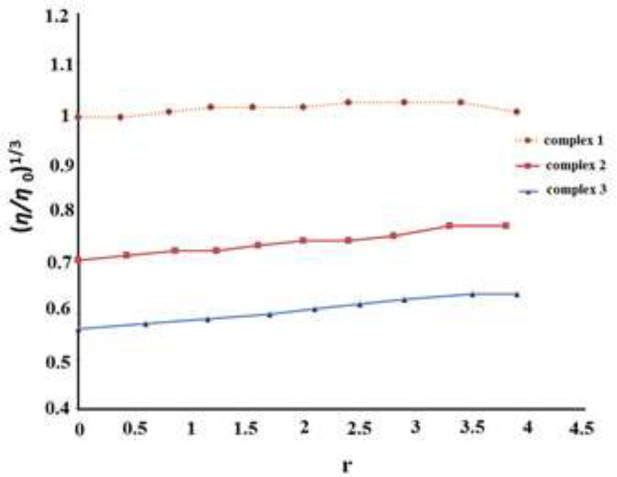
The effects of growing amounts of compounds **1**, **2 **and **3** on an FS-DNA viscosity at 298 K. The FS-DNA was concentration 1.4 × 10 ^−4^ mol L^−1^

In the absence of crystallographic data, viscosity measurement is an effective method for detecting changes in DNA conformation. In intercalative interaction, the dissociation of FS-DNA nucleotides for the insertion of ligand results in DNA length increase, which increases the DNA viscosity (42, 43) while in the case of non-intercalative interactions namely electrostatic and groove binding the viscosity of DNA will not alter. The comparative values of FS-DNA viscosity were obtained for different concentrations of synthesized complexes based on the equation η = (t – t_0_)/t _0_, where *t* refers to the flow time for sample solution and *t*_0_ is blank solution flow time, respectively. Figure 12 shows the comparative values of FS-DNA viscosity did not change with the growing concentration of complexes **1**,** 2 **and** 3** in an FS-DNA solution containing all the synthesized compounds. The outcomes prove a non-intercalative interaction mode.

*Circular Dichroism analysis*

CD spectroscopy provides complementary information to absorption spectra. This practical technique is sensitive and non-destructivethat used to analyze the conformations of optically active species such as nucleic acids in solution. In this study, CD spectroscopy data were applied for the detection of slight structural modification of DNA in the binding procedure (44, 45). 

Observed changes in signals are often linked to corresponding changes in DNA structure. Any change in DNA conformation causes a change in produced signals. The solution of the right-handed DNA helix makes a negative band near 220 nm and the positive band around 270-290 nm arises from base pairing and base stacking. The binding mode DNA-complex affects these bands (44, 46).

**Figure 13:**
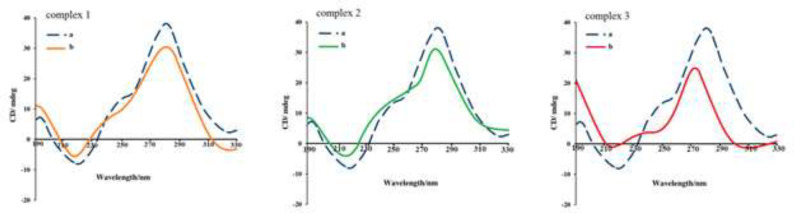
The CD spectra of DNA with increasing concentration of complexes **1,2 **and** 3**. The concentration of DNA was 1.4 × 10 ^−4^ mol.L ^−1^. [Complex] **/** [DNA] ratio were (a) 0.00, 0.23 and 0.042 respectively

It has been reported that intercalation increases base stacking and eventuates to the stabilization of B conformation of right-handed DNA and increases the intensity of both bands. On the other hand, DNA conformation is not significantly affected in the case of groove and electrostatic binding or non-intercalation (44). The effect of growing concentrations of Cr(III) complexes on DNA conformation has been reported as CD spectral data and the results are illustrated in Figure 13.

The CD changes at both wavelengths include a decrease in the magnitude of the bands and reduction of, proving non-intercalative interactions and also reduction of base stacking (42). Thus, the data suggests non-intercalation and confirms groove and electrostatic mode of binding.

*Agarose gel electrophoresis*

**Figure 14:**
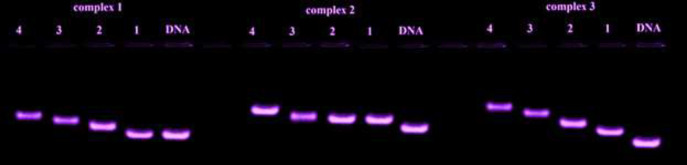
DNA fragments at different concentrations of complexes (**1**, **2** and **3**) at 25 °C: one lane having: DNA control; lane 1-4: complex (**1**, **2** and **3**) + FS-DNA

Agarose gel electrophoresis is a simple and highly efficient technique for identifying the interaction mode of complex-DNA. For this purpose, different concentrations of synthesized complexes were mixed with 4 μL of loading buffer, methylene blue and 5 μL DNA solution (1.4 × 10^-4^M). The solution was loaded on a prepared agarose gel and was run at a voltage of 100 v for a while to obtain the highest level of separation. Finally, the DNA fragments were visualized after irradiation with UV light. Figure 14 confirms the interaction of DNA and synthesized complexes.

*Determination of anti-proliferative activity*

The synthesized compounds** (1, 2 **and** 3) **were monitored by MTT assay toward two human cancer cell lines A549, KB and HUVEC as normal cells compared to that of cisplatin as a standard drug, and the results were reported in terms of IC_50_ values. As shown in Table 2 the compounds exhibited high potencies in the low micromolar range against cancer cell lines. The synthetic metal complexes [Cr(dafone)_2_(H_2_O)_2_](NO_3_)_3 _**(1)** (5.7 ± 0.2 and 4.0 ± 0.7 µM against A549 and KB) and [Cr(opd)(dafone)_2_](NO_3_)_3_ **(2) (**4.8 ± 0.1 and 3.4 ± 0.5 µM against A549 and KB) revealed a substantial decrease in the percentage of viable cells while [Cr(phen-dione)(dafone)(H_2_O)_2_](NO_3_)_3 _**(3) **exhibited a moderate activity as 39.0 ± 2.0 µM and 53.6 ± 2.8 µM against A549 and KB respectively. Cisplatin IC_50_ values as the positive control, against A549 and KB cancer cell lines, are 14.6 ± 0.6 and 6.2 ± 0.3 µM, respectively. Comparing the chemical structure of the target compounds indicates that the presence of dafone ligand could be the reason for better efficiency of **1 **and** 2 **rather than** 3. **The cytotoxicity evaluation against HUVEC cells exhibited about two to three times lower IC_50_ values for both synthesized compounds and cisplatin. The target values for **1** and **2** are 11.22 ± 0.9, 15.49 ± 1.0 while compound **3** did not show cytotoxicity against normal cells. The obtained value for cisplatin was 58.88 ± 3.5.

*Molecular docking results*

**Figure 15:**
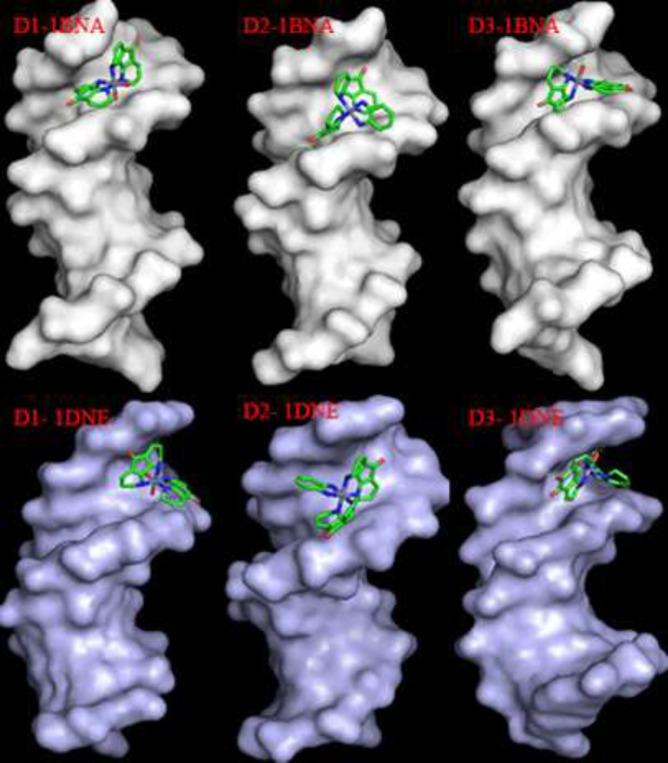
Docking pose of synthesized Cr(III) complexes **1**, **2** and **3** in DNA minor groove

The molecular docking simulation was performed to explore the mode of DNA-complex interaction. The most favorable poses with the minimum energy are illustrated in Table 3. The stabilization of synthesized complexes in binding to DNA occurs via different hydrogen bonds. The more negative binding energies indicate the higher binding affinity of the synthesized compound in the DNA binding site. As shown in Figure 15 all the synthesized complexes interact with DNA in a groove binding mode.

**Table 1:** Different thermodynamic parameters at 303, 298 and 293 K

**Complex**	**K (T)**	**K**_SV _ **× 10** ^5 ^ **(M** ^-1^ **)**	**k**_q _ **× 10** ^13 ^ **(M** ^-1^ **s** ^-1^ **)**	**n**	**K**_b _ **×** **10**^-5 ^**(M**^-1^**)**		**∆G** **˚ ** **(kJ/mol)**	**ΔH** **˚ ** **(kJ/mol)**	**ΔS** **˚ ** **(J/mol.K)**
					**Fluorescence**	**UV-Vis**			
**1**	303	2.75 ± 0.02	2.75 ± 0.02	1.00	1.4 ± 0.01		-33.73 ± 0.02		
	298	3.81 ± 0.01	3.81 ± 0.01	1.03	3.1 ± 0.02	5.1 ± 0.03	-31.63 ± 0.04	-93.53 ± 0.03	-0.42 ± 0.02
	293	4.36 ± 0.01	4.36 ± 0.01	1.04	4.4 ± 0.01		-29.53 ± 0.02		
**2**	303	3.95 ± 0.03	3.95 ± 0.03	1.04	2.4 ± 0.01		-45.34 ± 0.01		
	298	4.86 ± 0.01	4.86 ± 0.01	1.05	3.1 ± 0.03	1.2 ± 0.01	-42.24 ± 0.01	-142.52 ± 0.02	-0.62 ± 0.03
	293	8.35 ± 0.04	8.35 ± 0.04	1.07	5.1 ± 0.02		-39.14 ± 0.02		
**3**	303	2.32 ± 0.01	2.32 ± 0.01	1.09	3.9 ± 0.01		-14.70 ± 0.01		
	298	2.97 ± 0.03	2.97 ± 0.03	1.14	4.1 ± 0.01	3.5 ± 0.01	-13.60 ± 0.03	-51.96 ± 0.01	-0.22 ± 0.01
	293	4.57 ± 0.02	4.57 ± 0.02	1.16	4.4 ± 0.03		-12.50 ± 0.01		

**Table 2:** Anti-proliferative activity of synthesized Cr(III) complexes

**Compound**	^an ^ **IC**_50 _ **(µM)**		
	**A549**	**KB**	**HUVEC**
**1**	5.7 ± 0.2	4.0 ± 0.7	11.22 ± 0.9
**2**	4.8 ± 0.1	3.4 ±0.5	15.49 ± 1.0
**3**	39.0 ± 2.0	53.6 ± 2.8	>100
**Cisplatin**	14.6 ± 0.6	6.2 ± 0.3	58.88 ± 3.5

^ a^Data represent the mean value of at least three independent experiments in triplicate. (Mean ± SEM).

**Table 3:** Docking results of the synthesized Cr(III) complexes **1**, **2** and **3** in interaction with two different DNA Structures

Ligand	PDB ID	Binding energy (kJ.mol^-1^)	H-bonds	Adjacent nucleotides
**1**	**1BNA**	-277.14	H_2_O …Phosphate O (G16, 3.2Å)	A17, G16, C15, C11
			Dafone C=O… Phosphate O (A17, 3.4Å)	
	**1DNE**	-295.71	^1^H_2_O …backbone ribose O (C23, 2.6Å)	C23, C24C23, C24
			^1^H_2_O …NH (G22, 2.5Å)	
			^1^H_2_O …O=C (C3, 3.4Å)	
			^2^H_2_O …NH (G4, 3.0Å)	
			^2^H_2_O …Phosphate O (G4, 2.7Å)	
			^2^H_2_O …O=C (C3, 3.1Å)	
**2**	**1BNA**	-299.87	Dafone C=O… Phosphate O (A5, 2.6Å)	C3, C4, C23
			Dafone C=O… Phosphate O (A5, 2.7Å)	
	**1DNE**	-303.58	Dafone C=O… NH (G2, 3.4Å)	C3, C23, A5
			opd N… NH (G22, 3.4Å)	
			opd N… NH (G4, 3.4Å)	
**3**	**1BNA**	-284.63	Dafone C=O… Phosphate O (A18, 3.4Å)	A17, C15, G10
			^1^H_2_O …Phosphate O (C11, 3.1Å)	
			^2^H_2_O …Phosphate O (C11, 3.3Å)	
			^2^H_2_O … backbone ribose O (C23, 2.6Å)	
			Phen dione C=O… backbone ribose O (G16, 3.1Å)	
	**1DNE**	-293.18	Phen dione C=O… Phosphate O (G10, 2.8Å)	C15, A17
			^1^H_2_O …backbone ribose O (G10, 2.4Å)	
			^1^H_2_O … NH (G10, 3.2Å)	
			^1^H_2_O …Phosphate O (C11, 2.8Å)	
			^2^H_2_O …Phosphate O (C11, 2.9Å)	
			^2^H_2_O …Phosphate O (C11, 3.4Å)	
**Dafone C=O … Phosphate O (G16, 2.9Å)**

## Conclusion

Herein, we report the synthesis of three complexes of Chromium (III) with 4,5-Diazafluoren-9-one ligand and characterization of the complexes using FT-IR and UV–Visible spectroscopy, CV, and elemental analysis. The interactions between synthesized complexes with FS-DNA at physiological (pH = 7.2) were investigated through different spectroscopic methods and gel electrophoresis. UV–Vis spectra indicated that Cr(III) compounds interact with DNA strands showing appropriate values of binding constant. The fluorescence data show a static mechanism of quenching and reduction of K_b_ values with growing temperature indicates the exothermic interaction. Thermodynamic factors obtained from fluorescence data show that (negative values of *∆Hº* and *∆Sº*) the stabilization of metal complexes in DNA grooves occurs via van der Waals forces and hydrogen bonding. It was known that complexes **1**, **2** and **3** were bound to DNA with a strong affinity and binding reaction was mainly carried out using van der Waals forces and hydrogen bonding. Cytotoxicity assessment proposed that the synthesized compounds are potent anti-proliferative agents and could be further studied as anti-cancer agents. It was detected from the docking simulations that all the synthesized complexes bind via groove mode as confirmed by experimental outcomes. 
